# Association between Transient-Continuous Hypotension during Mechanical Thrombectomy for Acute Ischemic Stroke and Final Infarct Volume in Patients with Proximal Anterior Circulation Large Vessel Occlusion

**DOI:** 10.3390/jcm13133707

**Published:** 2024-06-25

**Authors:** Marcin Wiącek, Izabella Tomaszewska-Lampart, Marzena Dziedzic, Anna Kaczorowska, Halina Bartosik-Psujek

**Affiliations:** 1 Department of Neurology, Institute of Medical Sciences, University of Rzeszow, 35-959 Rzeszow, Poland; itomaszewska@ur.edu.pl (I.T.-L.); bartosikpsujek@op.pl (H.B.-P.); 2 Department of Neurology, Clinical Regional Hospital No. 2, 35-301 Rzeszow, Poland; marzena.dziedzic4@gmail.com (M.D.); annkacz98@gmail.com (A.K.)

**Keywords:** acute ischemic stroke, mechanical thrombectomy, hypotension, blood pressure, final infarct volume, large vessel occlusion

## Abstract

**Background/Objectives**: Periprocedural blood pressure changes in stroke patients with a large vessel occlusion are a known modifiable risk factor of unfavorable treatment outcomes. We aimed to evaluate the association between pre-revascularization hypotension and the final infarct volume. **Methods**: In our retrospective analysis, we included 214 consecutive stroke patients with an anterior circulation large vessel occlusion that underwent mechanical thrombectomy under general anesthesia. Noninvasively obtained blood pressure values prior to symptomatic vessel recanalization were analyzed as a predictor of post-treatment infarct size. Linear logistic regression models adjusted for predefined factors were used to investigate the association between blood pressure parameters and the final infarct volume. **Results**: In our cohort, higher baseline systolic blood pressure (aβ = 8.32, 95% CI 0.93–15.7, *p* = 0.027), its maximal absolute drop (aβ = 6.98, 95% CI 0.42–13.55, *p* = 0.037), and >40% mean arterial pressure decrease (aβ = 41.77, CI 95% 1.93–81.61, *p* = 0.040) were independently associated with higher infarct volumes. Similarly, continuous hypotension measured as intraprocedural cumulative time spent below either 100 mmHg (aβ = 3.50 per 5 min, 95% CI 1.49–5.50, *p* = 0.001) or 90 mmHg mean arterial pressure (aβ = 2.91 per 5 min, 95% CI 0.74–5.10, *p* = 0.010) was independently associated with a larger ischemia size. In the subgroup analysis of 151 patients with an M1 middle cerebral artery occlusion, two additional factors were independently associated with a larger ischemia size: systolic blood pressure maximal relative drop and >40% drop from pretreatment value (aβ = 1.36 per 1% lower than baseline, 95% CI 0.04–2.67, *p* = 0.043, and aβ = 43.01, 95% CI 2.89–83.1, *p* = 0.036, respectively). No associations between hemodynamic parameters and post-treatment infarct size were observed in the cohort of intracranial internal carotid artery occlusion. **Conclusions**: In patients with ischemic stroke due to a proximal middle cerebral artery occlusion, higher pre-thrombectomy treatment systolic blood pressure is associated with a larger final infarct size. In patients treated under general anesthesia, hypotension prior to the M1 portion of middle cerebral artery recanalization is independently correlated with the post-treatment infarct volume. In this group, every 5 min spent below the mean arterial pressure threshold of 100 mmHg is associated with a 4 mL increase in ischemia volume on a post-treatment NCCT. No associations between blood pressure and final infarct volume were present in the subgroup of patients with an intracranial internal carotid artery occlusion.

## 1. Introduction

Mechanical thrombectomy (MT) is considered the mainstay of large vessel occlusion (LVO) acute ischemic stroke treatment [1]. Despite nearly 90% successful reperfusion rates using modern stent retriever and aspiration devices, only half of the patients undergoing endovascular treatment (EVT) achieve functional independence [2]. Several factors affecting MT outcomes have been identified, with periprocedural hemodynamic parameters being of clinical importance due to their modifiable character [3]. Numerous studies have shown blood pressure (BP) variability, including intraprocedural hypotension, to be an independent risk factor for unfavorable clinical outcomes [4,5,6]. It is hypothesized that a lower systemic BP prior to symptomatic vessel recanalization limits collateral flow and perfusion of penumbral tissue, leading to larger post-intervention infarct volumes and poorer functional treatment results [7,8]. Recently, one study showed that intraprocedural hypotension can lead to serious EVT treatment complications, such as malignant brain edema and symptomatic intracranial hemorrhage, which may be further mediators of worse clinical outcomes [9].

General anesthesia has been shown to pose a greater risk of BP decrease compared to MT under conscious sedation [5,10]. It is postulated that episodes of blood pressure drops may be responsible for the worse clinical outcomes of EVT under GA indicated in some studies [11,12,13].

In this work, we assessed the effects of transient and continuous hypotension prior to symptomatic vessel recanalization on post-treatment infarct size in a homogenous cohort of patients undergoing MT under GA for anterior circulation LVO.

## 2. Materials and Methods

### 2.1. Study Design

This is an observational study based on the retrospective analysis of a single academic center prospective registry comprising consecutive adult (age ≥ 18 years) patients with acute ischemic stroke undergoing EVT. It was conducted at the academic comprehensive stroke center: Department of Neurology, Clinical Regional Hospital No. 2, Rzeszow, Poland. The study was approved by the local Bioethics Committee (no. 2022/50). Written patient consent was waived due to the observational status of the study with no patient intervention.

### 2.2. Patient Selection and Treatment Parameters

**Figure 1:**
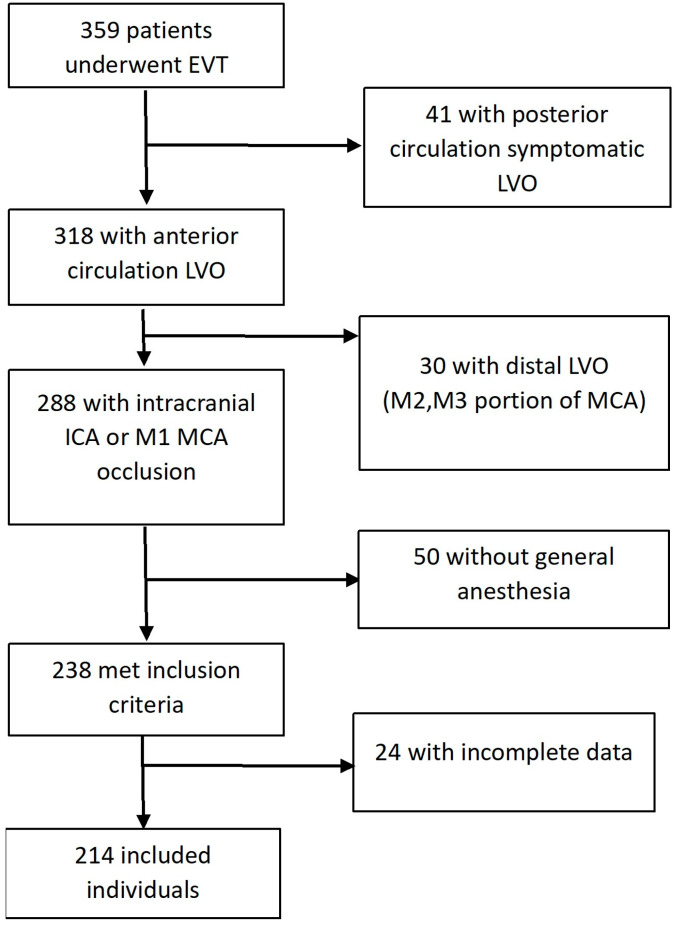
Flow chart of the inclusion of study population. EVT, endovascular treatment; LVO, large vessel occlusion; ICA, internal carotid artery; MCA, middle cerebral artery.

We included a consecutive sample of patients that underwent EVT over a period of 28 months (between 1 December 2018 and 31 March 2021) and met the following inclusion criteria: (a) age ≥ 18 years; (b) occlusion of the intracranial portion of the internal carotid artery (ICA) and/or proximal segment (M1) of the middle cerebral artery (MCA); and (c) EVT under general anesthesia. The flow chart of the inclusion of the study population is displayed in Figure 1.

Patient demographic data, medical history, baseline characteristics, and imaging data were obtained from electronic medical records. Stroke etiology was classified according to the TOAST (Trial of Org 10172 in Acute Stroke Treatment) classification. Localization of LVO was regarded as the most proximal intracranial occlusion, and tandem occlusion as a simultaneous extracranial ICA and intracranial artery closure.

All patients were treated with EVT using modern stent retrievers and/or direct aspiration technique. All treatment decisions were made by the endovascular treatment team (experienced neuroradiologist, vascular neurologist and anesthesiologist) in accordance with the Polish Neurological Society stroke treatment guidelines [14]. General anesthesia was the preferred anesthesia method according to the local protocol. Hypotension (SBP < 120 mmHg) was treated with ephedrine, phenylephrine, or norepinephrine, and hypertension (>180 mmHg) with urapidil at the anesthesia treatment team’s discretion.

### 2.3. Blood Pressure Data

Blood pressure data were obtained from the patient’s anesthesia report following the intervention. These data included systolic blood pressure (SBP), diastolic blood pressure (DBP), and mean blood pressure (MAP) measured non-invasively before the EVT procedure and every 5 min until LVO recanalization, leading to successful reperfusion (or termination of procedure in the case of unsuccessful reperfusion). We assessed the following BP parameters: (a) baseline SBP/DBP/MAP (defined as the value of the measurement directly preceding the induction of anesthesia); (b) intraprocedural BP decrease/increase below or over the given SBP/MAP threshold; (c) intraprocedural minimal/maximal SBP/MAP value; (d) intraprocedural maximal SBP/MAP drop below its baseline value (SBP/MAPmax. drop); (e) SBP/MAP max. drop > 0%/ > 20%/ > 40% (yes/no); (f) cumulative time spent below/over the given SBP/MAP threshold (to calculate the time over/below the threshold, a single BP measurement was treated as a continuous 5 min BP value, as described elsewhere [5]).

The BP thresholds were established a priori to define hypotension (SBP < 140/120/100 mmHg, MAP < 100/90/80 mmHg) and hypertension (SBP > /160/180 mmHg, MAP > 110/120 mmHg). The thresholds for relative hypotension (SBP/MAP max. drop) were set to >0/20/40% SBP/MAP drop from the baseline value.

### 2.4. Outcome Measurements

All patients underwent NCCT imaging as part of their routine clinical work-up 24–36 h post-EVT. The final infarct volume (FIV) was evaluated by two experienced vascular neurologists and calculated using a validated semi-automatic method (3D Slicer v.4.10) as described elsewhere [15]. In the case of discrepancies, ≤10% the mean of both values was calculated. If the difference was more than 10%, the final result was determined by consensus opinion. Post-MT reperfusion was graded by two experienced neuroradiologists using the Thrombolysis in Cerebral Infarction (TICI) scale: grades 2b (perfusion > 50% of the vascular distribution of the occluded artery) and 3 (complete perfusion with the filling of all distal branches) were considered as successful reperfusion [16].

### 2.5. Statistical Analyses

The analyzed variables were presented as the mean ± standard deviation or median with an interquartile range depending on the normality of the distribution (according to the Shapiro–Wilk test). Categorical variables were presented as numbers (percentage). Univariate analyses of ordinal and continuous variables with the FIV were performed using Spearman’s rank correlation coefficient. Associations between hemodynamic variables and the FIV were determined using linear logistic regression models adjusted for predefined factors, selected according to prior studies and theoretical considerations (age, pretreatment NIHSS score, onset-to-groin time, most proximal symptomatic vessel occlusion, presence of successful reperfusion [TICI 2b-3]). All statistics were computed using PQStat Software 1.8 (Poznan, Poland). Statistical significance was set at *p* < 0.05 (two-tailed).

## 3. Results

Two hundred and fourteen patients were included, with a median age of 71 years (IQR 65–79), 101 (47.2%) females, and a 17 ± 5 NIHSS score at admission. The etiology of ischemic stroke was cardiogenic in 119 (55.6%), due to large vessel atherosclerosis in 24 (11.2%), and an ICA dissection in 5 (2.4%) patients. One (0.5%) subject had another determined etiology (thrombophilia), and in 65 (30.4%) individuals, the stroke etiology was classified as undetermined (mainly due to two or more possible causes of stroke). Seven (3.2%) patients had pre-stroke disability (modified Rankin scale, mRS ≥ 3). A MAP decrease from its pretreatment value was observed in 208 (97.2%) individuals. Moreover, 171 (79.9%) patients experienced an intraprocedural MAP drop of more than 20%, and 124 (57.9%) of more than 40% compared to the baseline MAP. The median FIV on a post-treatment NCCT was 80.5 (IQR 20–204) mL. The baseline characteristics of the evaluated subjects are presented in Table 1.

**Figure 2:**
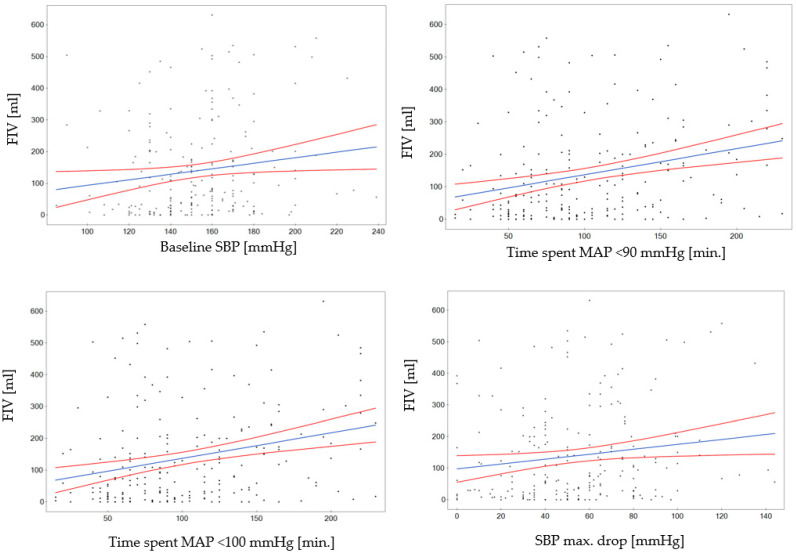
Association between hemodynamic parameters and the final infarct volume. Scatter plots based on linear regression models adjusted for predefined confounding factors. The last scatter plot shows analysis of the MCA M1 occlusion subgroup. FIV, final infarct volume; SBP, systolic arterial blood pressure; MAP, mean arterial blood pressure.

In our cohort, a higher value of the baseline SBP was associated with a larger infarct volume on NCCT 24–36 h after treatment (*p* = 0.014; Figure 2). A larger FIV was also positively correlated with the maximal intraprocedural absolute SBP drop from its pretreatment value (*p* = 0.016). A similar association was observed for the SBP relative decrease (percentage of baseline SBP measurement; *p* = 0.014). Among patients who experienced >40% SBP or >40% MAP drop prior to LVO recanalization, the FIV was significantly higher (median 128 vs. 56 mL, *p* = 0.010 for >40% SBP and 130 vs. 59, *p* = 0.005 for a >40% MAP drop). The univariate analyses of given hemodynamic parameters are presented in Table 2.

After adjusting for potential confounders, a higher baseline SBP (adjusted regression coeff. [aβ] = 8.32, 95% CI 0.93–15.7, *p* = 0.027), absolute SBP maximal drop (aβ = 6.98, 95% CI 0.42–13.55, *p* = 0.037), and >40% MAP drop from the baseline (aβ = 41.77, CI 95% 1.93–81.61, *p* = 0.040) were independently associated with a higher FIV. The multivariable analyses are presented in Table 3.

Continuous hypotension, measured as intraprocedural cumulative time spent below 100 mmHg MAP (*p* < 0.001), 90 mmHg MAP (*p* = 0.001), and 140 mmHg SBP (*p* = 0.033), was positively correlated with a higher FIV value. Time spent under 100 mmHg and 90 mmHg thresholds was independently associated with a larger infarct size after adjusting the linear regression models for predefined confounding factors (aβ = 3.50, 95% CI 1.49–5.50, *p* = 0.001 for 100 mmHg and aβ = 2.91, 95% CI 0.74–5.10, *p* = 0.010 for 90 mmHg threshold). Every 5 min of hypotension defined as MAP below 100 mmHg was associated with a 3.5 mL (1.49–5.5, 95% CI) larger infarct after MT treatment.

In the subgroup analysis of 151 patients with an M1 MCA occlusion, two additional factors were independently associated with larger infarct volumes: SBP maximal relative drop and its >40% drop from the pretreatment value (aβ = 1.36, 95% CI 0.04–2.67, *p* = 0.043, and aβ = 43.01, 95% CI 2.89–83.1, *p* = 0.036, respectively). No association was found between evaluated hemodynamic parameters and FIV in the subgroup of patients with an intracranial ICA occlusion. The MCA occlusion subgroup analyses were presented in Table 2 and Table 3.

The associations between hemodynamic parameters and the final infarct volume are presented in Figure 2.

## 4. Discussion

In our study, we demonstrated that both transient and continuous intraprocedural hypotension, as well as pretreatment hypertension, are independently associated with larger infarct volumes in a cohort of acute ischemic stroke patients undergoing MT for proximal MCA, but not an intracranial ICA occlusion.

It is postulated that a poor collateral status can influence systemic BP, with higher pre-treatment BP values promoting collateral flow and the perfusion of penumbral tissue [17,18]. The association between insufficient collaterals and an elevated BP in LVO patients was confirmed in some studies [19,20]. That indicates that the hypertensive reaction in acute ischemic stroke could possibly be only an epiphenomenon of poor collateral status, with the latter directly influencing the post-MT infarct size and clinical outcome. This could explain our finding that a higher baseline SBP correlates with a larger FIV after an EVT. The other mechanism of a larger FIV in terms of pretreatment hypertension could be the relative resistance to LVO recanalization, resulting in lower reperfusion rates that were found in patients with SBP >150 mmHg [17]. The hydromechanical force affecting the clot can hinder its retrieval and potentially lead to longer onset-to-reperfusion times. In our cohort, a higher baseline SBP was correlated with a larger FIV, and the effect was independent of recanalization time and reperfusion status.

We also found that intraprocedural pre-recanalization hypotension in patients with an M1 MCA occlusion, evaluated as the maximal SBP drop and large relative (>40%) MAP decrease, is independently associated with larger infarct volumes. Our results are consistent with previous work that found a link between intraprocedural hypotension during an MT, infarct growth, larger FIV, and poorer clinical outcomes [7]. This prior work confirms the detrimental effect of lowering blood pressure during an EVT, with several parameters found to increase the risk of unfavorable functional outcomes: maximal MAP fall, MAP drop of more than 10 or 40%, and average intraprocedural MAP > 10% lower than the baseline [7,21,22,23]. Together with our results, this can support the hypothesis of collateral flow restriction in terms of systemic BP reduction with faster infarct growth, its larger final size and, as a result, larger patient disability [7,24]. Additionally, the presence of intraprocedural SBP/MAP elevations of more than 120% than its baseline value was found to be an independent predictor of a post-MT smaller ischemia size in one study [25]. It was probably associated with avoiding procedural BP decreases that are strongly associated with poor clinical outcomes (and a larger FIV in our cohort), rather than hypertension causing better tissue outcomes itself.

Momentary hypotension parameters are probably only the surrogate markers of continuous intraprocedural BP decreases, which was also shown to influence functional MT outcomes. Rasmussen et al. recognized hypotension, defined as a MAP < 70 mmHg of more than 10 min in duration, to be an independent risk factor of unfavorable clinical outcomes (mRS > 2) [5]. Similarly, sustained hypotension (area between admission MAP and continuous measurements of intraprocedural MAP) has been found to affect patient prognosis [7]. Our study provides additional evidence of continuous BP lowering being an independent predictor of larger post-treatment ischemia volumes. Time spent below both 100 mmHg and 90 mmHg of MAP thresholds was associated with a larger FIV. Those thresholds are different than the values shown by Rasmussen et al., but in our cohort, time spent in predefined hypotension was of long enough duration to potentially influence the assessment results. Secondly, there is a chance that the thresholds for clinically and radiographically unfavorable outcomes differ. It is also worth noting that the intraprocedural period spent below predefined SBP thresholds was not independently associated with tissue outcomes. This may be explained by the fact that the MAP, rather than the SBP, affects the collateral perfusion pressure, maintaining penumbral blood supply and preventing its conversion to an infarct core [24]. This could point to the conclusion that careful fluid management and the use of vasopressors may halt the ischemic core volume progression [26,27].

In our cohort, only the subgroup of patients with the symptomatic M1 portion of an MCA occlusion showed a significant association with evaluated hemodynamic parameters. No significant correlations between intraprocedural hypotension duration and FIV were found in the subgroup of patients with an intracranial ICA occlusion. This could possibly be explained by the more common atherosclerotic etiology of ischemic stroke among those subjects (which can affect collateral formation), anomalies of the circle of Wills that can affect the final infarct size, and longer duration of procedure in those patients.

To our knowledge no data on the association between BP decreases during an MT and serious EVT complications have been presented.

A strength of our paper is that we showed both continuous and transient hypotension to affect the post-treatment infarct volume. Additionally, we found that only the subgroup of patients with a proximal MCA but not intracranial ICA show an association with intraprocedural blood pressure changes. To our knowledge, the latter finding has not been previously presented in the literature.

The limitations of our study include the retrospective, single center design, and modest sample size, with a potential for selection bias. Secondly, the continuous BP values were only an extension of intermittent non-invasive measurements, instead of continuous invasive BP assessment. Therefore, our results should be interpreted with caution.

## 5. Conclusions

In conclusion, in patients with ischemic stroke due to a proximal middle cerebral artery occlusion, a higher pre-MT treatment systolic blood pressure is associated with a larger final infarct size. In patients treated under general anesthesia, hypotension prior to the M1 portion of middle cerebral artery recanalization is independently correlated with the post-treatment infarct volume. The hemodynamic parameters that showed this association are intraprocedural time spent with <100 mmHg and <90 mmHg, as well as relative SBP decrease below its baseline value and a large >40% MAP drop. Every 5 min spent below the mean arterial pressure threshold of 100 mmHg is associated with a 4 mL increase in ischemia volume on a post-treatment NCCT. No associations between blood pressure and the final infarct volume were present in the subgroup of patients with an intracranial internal carotid artery occlusion.

## Figures and Tables

**Table 1:** Baseline characteristics and imaging treatment outcomes.

Total Patients	214
Age, years (median, IQR)	71 (65–79)
Female, N (%)	101 (47.2)
**Medical history**	
Hypertension, N (%)	175 (81.8)
Hyperlipidemia, N (%)	82 (38.3)
Atrial fibrillation, N (%)	121 (56.5)
Diabetes mellitus, N (%)	51 (23.8)
Chronic heart failure, N (%)	41 (19.2)
Coronary artery disease, N (%)	39 (18.2)
Smoker, N (%)	18 (8.4)
Cancer, N (%)	6 (2.8)
**Baseline characteristics**	
Baseline NIHSS (mean ± SD)	17 ± 5
Onset-to-groin time, minutes (median, IQR)	256 (210–300)
Onset-to-reperfusion time, minutes (median, IQR)	325 (280–376)
Bridging thrombolysis, N (%)	154 (72)
Localization of occlusion, N (%)	
ICA	63 (29.4)
MCA M1	151 (70.6)
Tandem occlusion	62 (29)
**Imaging treatment outcome measures**	
TICI, N (%)	
0	13 (6.1)
1	9 (4.2)
2a	10 (4.7)
2b	67 (31.3)
3	115 (53.7)
Intracranial hemorrhage, N (%)	
HI1	25 (11.7)
HI2	18 (8.4)
PH1	12 (5.6)
PH2	27 (12.6)
Symptomatic intracranial hemorrhage, N (%)	23 (10.7)
Malignant brain edema, N (%)	55 (25.2)

ICA, internal carotid artery; MCA, middle cerebral artery.

**Table 2:** Univariate analyses of hemodynamic variable association with the final infarct volume.

	All Subjects			MCA M1 *		
**Hemodynamic Variable**	**Median (IQR)**	**Spearman R**	***p*-Value**	**Median (IQR)**	**Spearman R**	***p*-Value**
Baseline SBP, mmHg	150 (135–170)	0.17	**0.014**	150 (135–167)	0.21	**0.010**
Baseline MAP, mmHg	106 (97–118)	0.10	0.138	107 (97–117)	0.95	0.25
**Intraprocedural momentary BP values**						
SBP_min._, mmHg	100 (90–105)	−0.11	0.103	100 (90–105)	−0.10	0.21
MAP_min._, mmHg	160 (145–170)	−0.07	0.305	71 (65–78)	−0.8	0.32
SBP_max._, mmHg	160 (145–170)	0.11	0.125	160 (145–170)	0.11	0.17
MAP_max._, mmHg	113 (103–123)	0.05	0.440	113 (103–121)	0.04	0.66
SBP_max. drop_, mmHg	51 (35–74)	0.16	**0.016**	50 (34–70)	0.20	**0.012**
MAP_max. drop_, mmHg	36 (22–50)	0.11	0.115	35 (23–48)	0.13	0.11
SBP max. drop, %	35 (25–44)	0.17	**0.014**	35 (25–43)	0.20	**0.013**
>0%			0.518 *			0.518 *
>20%			0.151 *			0.151 *
>40%			0.010 *			0.010 *
MAP max. drop, %	34 (22–42)	0.11	0.094	34 (23–41)	0.14	0.089
>0%			0.158 *			0.158 *
>20%			0.727 *			0.727 *
>40%			0.005 *			0.005 *
**Intraprocedural continuous BP measures**						
Time over SBP [mmHg] threshold, minutes						
<140	50 (30–80)	0.15	**0.033**	45 (30–75)	0.14	0.092
<120	25 (10–50)	0.04	0.531	25 (10–45)	0.06	0.443
<100	0 (0–10)	0.11	0.125	0 (0–10)	0.06	0.441
>160	0 (0–5)	0.07	0.343	0 (0–5)	0.06	0.466
Time over MAP [mmHg] threshold, minutes						
<100	90 (65–135)	0.27	**<0.001**	85 (60–125)	0.25	**0.002**
<90	65 (40–95)	0.22	**0.001**	60 (40–90)	0.27	**<0.001**
<80	20 (5–45)	0.06	0.382	20 (5–45)	0.07	0.373
>110	5 (0–5)	0.05	0.467	5 (0–5)	0.01	0.923

Bold font indicates statistical significance. MCA, middle cerebral artery; IQR, interquartile range; SBP, systolic arterial blood pressure; MAP, mean arterial blood pressure. * Mann–Whitney test was used to evaluate the association between the presence of relative SBP/MAP drop over threshold and final infarct volume. Other calculations were made using the Spearman test.

**Table 3:** Multivariate linear regression analyses for final infarct volume.

	All Subjects			MCA M1 *		
Hemodynamic Variable	B	CI 95%	*p*-Value	B	CI 95%	*p*-Value
Baseline SBP, mmHg	8.32	0.93–15.7	**0.027**	1.24	0.42–2.07	**0.004**
**Intraprocedural Momentary BP Values**						
SBP max. drop, 10 mmHg	6.98	0.42–13.55	**0.037**	7.93	1.29–14.57	**0.019**
SBP max. drop, %	1.21	(−0.09)–2.5	0.066	1.36	0.04–2.67	**0.043**
>40% SBP drop	39.03	(−0.46)–78.54	0.053	43.01	2.89–83.1	**0.036**
>40% MAP drop	41.77	1.93–81.61	**0.040**	47.09	6.74–87.43	**0.022**
**Intraprocedural Continuous BP Measures**						
Time < 140 mmHg SBP, 5 min	0.38	(−0.14)–0.85	0.157	0.46	(−0.043)–0.96	0.073
Time below MAP threshold, 5 min						
<100 mmHg	3.50	1.49–5.50	**0.001**	3.96	1.97–5.95	**<0.001**
<90 mmHg	2.91	0.74–5.10	**0.010**	3.90	1.20–5.57	**0.002**

Bold font indicates statistical significance. For the analysis of each parameter’s association with FIV, the linear regression model was adjusted for age, reperfusion status, NIHSS at admission, onset-to-groin time, and site of most proximal occlusion. * The linear regression model for M1 MCA subgroup assessment was adjusted for age, reperfusion status, NIHSS at admission, and onset-to-groin time. MCA, middle cerebral artery; SBP, systolic arterial blood pressure; MAP, mean arterial blood pressure.

## Data Availability

Data are contained within the article.

## References

[1] Turc G., Bhogal P., Fischer U., Khatri P., Lobotesis K., Mazighi M., Schellinger P.D., Toni D., de Vries J., White P. (2019). European stroke organisation (ESO)—European Society for Minimally Invasive Neurological Therapy (ESMINT) guidelines on mechanical Thrombectomy in acute ischemic stroke. J. Neurointerv. Surg..

[2] Goyal M., Menon B.K., Van Zwam W.H., Dippel D.W.J., Mitchell P.J., Demchuk A.M., Dávalos A., Majoie C.B.L.M., Van Der Lugt A., De Miquel M.A. (2016). Endovascular thrombectomy after large-vessel ischaemic stroke: A meta-analysis of individual patient data from five randomised trials. Lancet.

[3] Tomaszewska-Lampart I., Wiącek M., Bartosik-Psujek H. (2022). Risk factors for infarct growth and haemorrhagic or oedematous complications after endovascular treatment—A literature review. Neurol. Neurochir. Pol..

[4] Malhotra K., Goyal N., Katsanos A.H., Filippatou A., Mistry E.A., Khatri P., Anadani M., Spiotta A.M., Sandset E.C., Sarraj A. (2020). Association of Blood Pressure With Outcomes in Acute Stroke Thrombectomy. Hypertension.

[5] Rasmussen M., Schönenberger S., Hendèn P.L., Valentin J.B., Espelund U.S., Sørensen L.H., Juul N., Uhlmann L., Johnsen S.P., Rentzos A. (2020). Blood Pressure Thresholds and Neurologic Outcomes After Endovascular Therapy for Acute Ischemic Stroke: An Analysis of Individual Patient Data From 3 Randomized Clinical Trials. JAMA Neurol..

[6] Huang X., Guo H., Yuan L., Cai Q., Zhang M., Zhang Y., Zhu W., Li Z., Yang Q., Zhou Z. (2021). Blood pressure variability and outcomes after mechanical thrombectomy based on the recanalization and collateral status. Ther. Adv. Neurol. Disord..

[7] Petersen N.H., Ortega-Gutierrez S., Wang A., Lopez G.V., Strander S., Kodali S., Silverman A., Zheng-Lin B., Dandapat S., Sansing L.H. (2019). Decreases in Blood Pressure During Thrombectomy Are Associated With Larger Infarct Volumes and Worse Functional Outcome. Stroke.

[8] Raychev R., Liebeskind D.S., Yoo A.J., Rasmussen M., Arnaudov D., Brown S., Saver J., Simonsen C.Z. (2020). Physiologic predictors of collateral circulation and infarct growth during anesthesia: Detailed analyses of the GOLIATH trial. J. Cereb. Blood Flow Metab..

[9] Wiącek M., Szymański M., Walewska K., Bartosik-Psujek H. (2022). Blood Pressure Changes During Mechanical Thrombectomy for Acute Ischemic Stroke Are Associated With Serious Early Treatment Complications: Symptomatic Intracerebral Hemorrhage and Malignant Brain Edema. Front. Neurol..

[10] Jagani M., Brinjikji W., Rabinstein A.A., Pasternak J.J., Kallmes D.F. (2016). Hemodynamics during anesthesia for intra-arterial therapy of acute ischemic stroke. J. Neurointerv. Surg..

[11] Berkhemer O.A., Berg L.A.v.D., Fransen P.S., Beumer D., Yoo A.J., Lingsma H.F., Schonewille W.J., Berg R.v.D., Wermer M.J., Boiten J. (2016). The effect of anesthetic management during intra-arterial therapy for acute stroke in MR CLEAN. Neurology.

[12] Campbell B.C.V., van Zwam W.H., Goyal M., Menon B.K., Dippel D.W.J., Demchuk A.M., Bracard S., White P., Dávalos A., Majoie C.B.L.M. (2018). Effect of general anaesthesia on functional outcome in patients with anterior circulation ischaemic stroke having endovascular thrombectomy versus standard care: A meta-analysis of individual patient data. Lancet Neurol..

[13] Davis M.J., Menon B.K., Baghirzada L.B., Campos-Herrera C.R., Goyal M., Hill M.D., Archer D.P., Program T.C.S. (2012). Anesthetic management and outcome in patients during endovascular therapy for acute stroke. Anesthesiology.

[14] Błażejewska-Hyżorek B., Czernuszenko A., Członkowska A., Ferens A., Gąsecki D., Kaczorowski R., Karaszewski B., Karliński M., Kaźmierski R., Kłysz B. (2019). Wytyczne postepowania w udarze mózgu. Pol. Prz. Neurol..

[15] Boers A.M., Marquering H.A., Jochem J.J., Besselink N., Berkhemer O., van der Lugt A., Beenen L., Majoie C. (2013). Automated cerebral infarct volume measurement in follow-up noncontrast CT scans of patients with acute ischemic stroke. AJNR Am. J. Neuroradiol..

[16] Hacke W., Kaste M., Fieschi C., von Kummer R., Davalos A., Meier D., Larrue V., Bluhmki E., Davis S., Donnan G. (1998). Randomised double-blind placebo-controlled trial of thrombolytic therapy with intravenous alteplase in acute ischaemic stroke (ECASS II). second European-Australasian acute stroke study investigators. Lancet.

[17] Nogueira R.G., Liebeskind D.S., Sung G., Duckwiler G., Smith W.S. (2009). Predictors of good clinical outcomes, mortality, and successful revascularization in patients with acute ischemic stroke undergoing thrombectomy: Pooled analysis of the mechanical Embolus removal in cerebral ischemia (MERCI) and Multi MERCI trials. Stroke.

[18] De Georgia M., Bowen T., Duncan K.R., Chebl A.B. (2023). Blood pressure management in ischemic stroke patients undergoing mechanical thrombectomy. Neurol. Res. Pract..

[19] Wufuer A., Mijiti P., Abudusalamu R., Dengfeng H., Jian C., Jianhua M., Xiaoning Z. (2019). Blood pressure and collateral circulation in acute ischemic stroke. Herz.

[20] Sim J.E., Chung J.W., Seo W.K., Bang O.Y., Kim G.-M. (2022). Association of Systolic Blood Pressure and Cerebral Collateral Flow in Acute Ischemic Stroke by Stroke Subtype. Front. Neurol..

[21] Löwhagen Hendén P., Rentzos A., Karlsson J.E., Rosengren L., Sundeman H., Reinsfelt B., Ricksten S.-E. (2015). Hypotension during Endovascular Treatment of Ischemic Stroke Is a Risk Factor for Poor Neurological Outcome. Stroke.

[22] Treurniet K.M., Berkhemer O.A., Immink R.V., Lingsma H.F., der Stam V.M.C.W.-V., Hollmann M.W., Vuyk J., van Zwam W.H., van der Lugt A., van Oostenbrugge R.J. (2018). A decrease in blood pressure is associated with unfavorable outcome in patients undergoing thrombectomy under general anesthesia. J. Neurointerv. Surg..

[23] Whalin M.K., Halenda K.M., Haussen D.C., Rebello L., Frankel M., Gershon R., Nogueira R. (2017). Even Small Decreases in Blood Pressure during Conscious Sedation Affect Clinical Outcome after Stroke Thrombectomy: An Analysis of Hemodynamic Thresholds. AJNR Am. J. Neuroradiol..

[24] Regenhardt R.W., Das A.S., Stapleton C.J., Chandra R.V., Rabinov J.D., Patel A.B., Hirsch J.A., Leslie-Mazwi T.M. (2017). Blood Pressure and Penumbral Sustenance in Stroke from Large Vessel Occlusion. Front. Neurol..

[25] Pikija S., Trkulja V., Ramesmayer C., Mutzenbach J.S., Killer-Oberpfalzer M., Hecker C., Bubel N., Füssel M.U., Sellner J. (2018). Higher Blood Pressure during Endovascular Thrombectomy in Anterior Circulation Stroke Is Associated with Better Outcomes. J. Stroke.

[26] La Via L., Vasile F., Perna F., Zawadka M. (2024). Prediction of fluid responsiveness in critical care: Current evidence and future perspective. Trends Anaesth. Crit. Care.

[27] Karamchandani K., Dave S., Hoffmann U., Khanna A.K., Saugel B. (2023). Intraoperative arterial pressure management: Knowns and unknowns. Br. J. Anaesth..

