# Correction: Wiącek et al. Association between Transient-Continuous Hypotension during Mechanical Thrombectomy for Acute Ischemic Stroke and Final Infarct Volume in Patients with Proximal Anterior Circulation Large Vessel Occlusion. *J. Clin. Med.* 2024, *13*, 3707

**DOI:** 10.3390/jcm14041261

**Published:** 2025-02-14

**Authors:** Marcin Wiącek, Izabella Tomaszewska-Lampart, Marzena Dziedzic, Anna Kaczorowska, Halina Bartosik-Psujek

**Affiliations:** 1 Department of Neurology, Institute of Medical Sciences, University of Rzeszow, 35-959 Rzeszow, Poland; itomaszewska@ur.edu.pl (I.T.-L.); bartosikpsujek@op.pl (H.B.-P.); 2 Department of Neurology, Clinical Regional Hospital No. 2, 35-301 Rzeszow, Poland; marzena.dziedzic4@gmail.com (M.D.); annkacz98@gmail.com (A.K.)

## Error in Table 3

In the original publication [1], there was a mistake in Table 3 as published. The results of the statistical analyses for SBP maximal drop [%], >40% SBP drop, and >40% MAP drop were reported incorrectly. Additionally, the table title incorrectly referred to the statistical analysis that was conducted. The corrected Table 3 appears below. The authors state that the scientific conclusions are unaffected. This correction was approved by the Academic Editor. The original publication has also been updated.

**Table 3:** Multivariate linear regression analyses for final infarct volume.

	All Subjects			MCA M1 *		
Hemodynamic Variable	B	CI 95%	*p*-Value	B	CI 95%	*p*-Value
Baseline SBP, mmHg	8.32	0.93–15.7	**0.027**	1.24	0.42–2.07	**0.004**
**Intraprocedural Momentary BP Values**						
SBP max. drop, 10 mmHg	6.98	0.42–13.55	**0.037**	7.93	1.29–14.57	**0.019**
SBP max. drop, %	1.21	(−0.09)–2.5	0.066	1.36	0.04–2.67	**0.043**
>40% SBP drop	39.03	(−0.46)–78.54	0.053	43.01	2.89–83.1	**0.036**
>40% MAP drop	41.77	1.93–81.61	**0.040**	47.09	6.74–87.43	**0.022**
**Intraprocedural Continuous BP Measures**						
Time < 140 mmHg SBP, 5 min	0.38	(−0.14)–0.85	0.157	0.46	(−0.043)–0.96	0.073
Time below MAP threshold, 5 min						
<100 mmHg	3.50	1.49–5.50	**0.001**	3.96	1.97–5.95	**<0.001**
<90 mmHg	2.91	0.74–5.10	**0.010**	3.90	1.20–5.57	**0.002**

Bold font indicates statistical significance. For the analysis of each parameter’s association with FIV, the linear regression model was adjusted for age, reperfusion status, NIHSS at admission, onset-to-groin time, and site of most proximal occlusion. * The linear regression model for M1 MCA subgroup assessment was adjusted for age, reperfusion status, NIHSS at admission, and onset-to-groin time. MCA, middle cerebral artery; SBP, systolic arterial blood pressure; MAP, mean arterial blood pressure.
